# Amino Acid Trp: The Far Out Impacts of Host and Commensal Tryptophan Metabolism

**DOI:** 10.3389/fimmu.2021.653208

**Published:** 2021-06-04

**Authors:** Heather M. Grifka-Walk, Brittany R. Jenkins, Douglas J. Kominsky

**Affiliations:** Department of Microbiology and Immunology, Montana State University, Bozeman, MT, United States

**Keywords:** tryptophan, kynurenine, indole, microbiome & dysbiosis, mucosal immmunity, aryl hydrocarbon receptor, serotonin

## Abstract

Tryptophan (Trp) is an essential amino acid primarily derived from the diet for use by the host for protein synthesis. The intestinal tract is lined with cells, both host and microbial, that uptake and metabolize Trp to also generate important signaling molecules. Serotonin (5-HT), kynurenine and its downstream metabolites, and to a lesser extent other neurotransmitters are generated by the host to signal onto host receptors and elicit physiological effects. 5-HT production by neurons in the CNS regulates sleep, mood, and appetite; 5-HT production in the intestinal tract by enterochromaffin cells regulates gastric motility and inflammation in the periphery. Kynurenine can signal onto the aryl hydrocarbon receptor (AHR) to elicit pleiotropic responses from several cell types including epithelial and immune cells, or can be further metabolized into bioactive molecules to influence neurodegenerative disease. There is a remarkable amount of cross-talk with the microbiome with regard to tryptophan metabolites as well. The gut microbiome can regulate the production of host tryptophan metabolites and can use dietary or recycled trp to generate bioactive metabolites themselves. Trp derivatives like indole are able to signal onto xenobiotic receptors, including AHR, to elicit tolerogenic effects. Here, we review studies that demonstrate that tryptophan represents a key intra-kingdom signaling molecule.

## Introduction

The intestinal mucosa provides crucial metabolic functions and forms a barrier that protects host tissue from foreign luminal content and pathogens. Intestinal epithelial cells (IECs) help form and maintain the barrier between the luminal space and immune, nervous, and circulatory system components on the basolateral side. Though it might seem that the mucosa defends against an army of outsiders, mucosal defense depends upon “peacekeeping” molecules derived from the diet or commensal microbes. It is well-supported that the gut microbiome and diet each influence host metabolism, homeostatic processes, and inflammatory responses, and many of these functions are modulated to a large extent by small molecule metabolites (1–4). There is currently much interest in identifying beneficial metabolites, their source, and the mechanism(s) by which they elicit protective host responses. The amino acid tryptophan (Trp) is a key cross-kingdom source of critical downstream metabolites. Host cells metabolize dietary Trp into kynurenine (Kyn) and its derivatives, 5-hydroxytryptophan (5-HT, serotonin) and tryptamine. Microbes recycle free Trp to build proteins, but also to generate poly-aromatic hydrocarbon compounds and indole-containing compounds (5, 6). Some Trp metabolites, both host- and microbe-derived, modulate intestinal mucosal function as well as extra-intestinal tissue physiology through direct binding to the host transcription factor aryl hydrocarbon receptor (AHR) (7–9). Ligand biding to AHR leads to an array of responses in various tissues. Here, we review Trp uptake and metabolism by host and microbiome and its role in maintaining mucosal tissue homeostasis and modulation of host inflammatory responses, as well as the role of these metabolites in the modulation of extra-intestinal tissue function.

## Trp Metabolism by Host and Microbes

Most Trp found in the human gut is diet-derived. Trp is an essential amino acid and thus is unable to be produced by most animals. Some commensal bacterial species can generate Trp *de novo*, however the overall contribution of these microbes to the Trp pool in the intestinal tract is limited. Dietary Trp enters the intestinal tract and reaches its highest relative concentration in the distal colon, where most proteolytic metabolism occurs (10). While the recommended amount of Trp for adults is 3.5-6 mg/kg/day, or 250-425 mg daily intake, adults ingest upwards of 1000 mg of Trp each day (11, 12). Nonetheless, circulating and stored Trp concentrations are the lowest among all amino acids, possibly indicating fast incorporation into these metabolic and protein synthesis pathways. Several HPLC (and UHPLC)-MS/MS-based analytical methods have been developed for detecting Trp and its metabolites in serum, intestinal tissue, stool, urine, and cerebral spinal fluid (13–17). Using stable isotope labeled standards to minimize matrix effects during sample preparation, Trp concentrations in healthy adult human serum fall in the range of 1,000–50,000 ng/mL (40-100 µM) and 100–5,000 ng/mL for Kyn (1.2-2 µM), with other serum Trp metabolites typically falling in the 1-100 nM range (15, 18). Lower Trp concentrations are associated with many diseases, neurologic and mood disorders including inflammatory bowel disease (IBD), chronic pain, and depression (11, 12, 15, 18, 19). In some cases, the [Kyn]/[Trp] ratio is used as a biomarker for inflammatory and psychiatric disorders, yet this approach remains controversial due to a lack of understanding of the underlying cause(s) for reduced Trp, whether it be reduced dietary Trp, malabsorption, or alterations in Trp metabolism (e.g. conversion to downstream metabolites like Kyn or microbial derivatives) (20, 21). There is a growing appreciation for the range of responses to Trp metabolites that contribute to human health, making it important to also consider the concentrations of a variety of Trp metabolites. Human stool samples and collection of various tissues from animal models have shed more light into the complex nature of Trp metabolism, local concentrations and relative abundances of Trp derivatives, and mechanistic insights into health and disease states (16, 20, 22).

### Host Uptake and Metabolism of Trp

Trp uptake in the intestine occurs *via* transport system proteins. Transporters that facilitate neutral amino acid influx and efflux include solute carrier proteins LAT1-4, B^0^AT1, and TAT1 (23). Epithelial cells and other intestinal cells break down Trp into metabolites to be utilized by the gastrointestinal tract. Some Trp is transported into the bloodstream for metabolism at other sites including the liver and CNS (24). Circulating Trp can enter tissues *via* neutral or aromatic amino acid transporter proteins located on endothelial cells. Once inside a cell, Trp is either 1) recycled for peptide synthesis, 2) converted into indole ring-containing amines (i.e. 5-HT, melatonin, N-acetyl-5-HT), or 3) shunted into the Kyn pathway. Interestingly, a small amount the Trp metabolite tryptamine is produced by hosts and intestinal microbes, and can engage both AHR and 5-HT receptors (25).

A small proportion of Trp (estimated to be about 5% of that not used for protein synthesis) is converted into serotonin (5-HT) (23). 5-HT is a neurotransmitter that regulates gut motility and secretion in the intestine and serves an important role in the CNS by controlling mood and physiological activities including sleep and appetite. In order to produce 5-HT, free Trp is transported into the cell and converted to 5-hydroxytryptophan by a tryptophan hydroxylase (TPH) enzyme, then into 5-HT by aromatic L-amino acid decarboxylase. Overwhelmingly, most physiological 5-HT is produced in the intestinal tract by enterochromaffin cells by the enzyme TPH1 to act locally or to be carried by platelets to distal sites including the liver, bone, and cardiovascular system. 5-HT is unable to cross the blood brain barrier but is indispensable for normal central and peripheral nervous system function (26). A relatively small but critical amount of 5-HT is produced by neuron-derived TPH2 in the CNS and periphery to act locally. The responses exhibited by different cells and tissues to 5-HT depends on the receptor expressed by that cell. For example, the receptor 5-HT_7_ has been described as anti-inflammatory due to its impacts on intestinal dendritic cells, while 5-HT_4_ elicits a proinflammatory response from IECs (27). On the other hand, 5-HT_4_ receptor engagement on neurons is neuroprotective and can promote neurogenesis (28). Extracellular 5-HT availability is regulated by the transport protein SERT (SLC6A4), which specifically imports 5-HT into the cell to be oxidized into 5-hydroxyindole-3-acetic acid. Since 5-HT can have diverse and potent effects on a wide number of cell types and tissues depending on the 5-HT receptor expression profile, control of available 5-HT receptor ligand *via* SERT is a primary method of managing serotonin metabolism. SERT is downregulated in mouse models of colitis as well as in human patients with irritable bowel syndrome (IBS) and IBD, and SERT-deficient mice are highly susceptible to colitis models (29–31). Altogether, Trp metabolism into the small molecule 5-HT is a tightly regulated process that has substantial effects in the GI tract as well as the CNS.

The majority of free, non-protein-building Trp is metabolized by the host through the Kyn pathway. The enzymes indolamine 2, 3-dioxygenase (encoded by *IDO1* or *IDO2*) and Trp 2,3-dioxygenase (encoded by *TDO2*) perform the rate-limiting step in the conversion of Trp to the AHR ligand, Kyn (32). These enzymes use oxygen (O_2_) to break the carbon-carbon double bond at the 2, 3 position of the indole ring of L-Trp to form N-formylkynurenine in the first step of IDO/TDO-mediated Trp metabolism (33). Transcription of IDO and TDO is tissue-specific. Most TDO is produced in the liver to metabolize circulating Trp; however, TDO production in the brain may have an impact in the development and function of the central nervous system (34, 35). IDO1, on the other hand, is widely expressed and highly responsive to inflammation. At baseline, IDO1 is responsible for homeostatic levels of Kyn and its downstream metabolites, which ultimately include quinolinic acid, kynurenic acid, NADH and niacin. In the setting of inflammation, and especially in the presence of interferons, IDO1 is upregulated in many cell types to metabolize Trp (and bind to Trp mimetics) to limit available Trp. IDO-mediated Trp metabolism is a key, long-understood mechanism of pathogen control that limits an essential amino acid to decrease pathogen fitness (36–39). This is one direct mechanism of pathogen control that is regulated by the interferon response. Although IDO and TDO are upregulated in response to the innate immune response, their activity subdues inflammatory processes *via* Trp limitation or generation of bioactive metabolites like Kyn and NADH. IDO expression by dendritic cells during inflammation controls extracellular Trp levels. In T cells, decreased Trp sets off a stress response to limit T cell activation and proliferation and promote suppressive and stable regulatory T (Treg) cell polarization (40, 41). Due to the mostly anti-inflammatory and cell-extrinsic effects of IDO1, its expression has been described as problematic in the setting of many cancers. Several tumor studies have demonstrated IDO1 upregulation that inhibits the anti-tumor activity of innate and adaptive immune cells. The activity and immune-suppressive function of this enzyme can be inhibited with competitive Trp mimetics, which is in early stages of exploration for cancer treatment (42–45).

### Microbial Uptake and Metabolism of Trp

Trp metabolism by microbial enzymes has been shown to have an extensive impact on the host (46). The evolutionary benefit provided to the microbes generating these molecules is unclear, but it has been speculated that the generation of modified Trp derivatives can provide electron acceptors that offset fermentation processes in an anaerobic environment (47). Additionally, indole production has been shown to be beneficial for bacterial quorum sensing, motility, antibiotic resistance, biofilm production, and defense against non-indole producers (48–51). In *E. coli*, Trp is imported *via* TnaB and converted into indole *via* the tryptophanase TnaA. The amount of indole produced by *E. coli* is limited simply by the amount of free Trp, which could explain why human fecal indole concentrations are relatively high, ranging from high µM to low mM concentrations (52, 53). In other bacteria, Trp and indole can be modified by other enzymes like aromatic amino acid aminotransferase and variations of indoleacetate/indoleacetaldehyde dehydrogenase/dehydratase enzymes that covert Trp to different secondary, tertiary, and quaternary metabolites, which include indole pyruvate, indole acetate, indole-3-carboxaldehyde, indole-3-propionate, indole acrylate, skatole, indirubin, and indigo (47, 54–56). Intestinal microbes are also able to produce small amounts of the neurotransmitter tryptamine (57). Many of these metabolites have been shown to be beneficial to the host as biologically active signaling molecules recognized by host xenobiotic receptors.

The microbiome can also influence host Trp metabolism. Commensal bacteria produce short chain fatty acids such as butyrate from dietary fiber to promote expression of the gene encoding TPH1 by enterochromaffin cells in the intestinal tract, delivering a profound impact on intestinal 5-HT production and Trp metabolism (58). Studies have shown that germ-free mice, which lack any microbiome, have a reduced ratio of Kyn to Trp presumably due unmetabolized free Trp and low expression of IDO; IDO and Kyn levels are normalized upon colonization with a conventional microbiome or probiotics (59–62). Altogether, germ-free mice have lower levels of Kyn, 5-HT and microbe-derived Trp metabolites in serum, which suggests that microbial influence on Trp metabolites can be pervasive and alter circulating metabolites to exert effects outside the intestine (63).

Given that intestinal microbes produce metabolites that can signal to the host outside of the intestine, dietary intervention and prebiotics or probiotics to increase beneficial Trp metabolite production would be attractive alternative therapies. In mouse models, *Lactobacillus* strains that are capable of metabolizing Trp are protective in IBD and metabolic syndrome models (64, 65). Administration of *Bifidobacteria infantis* to rats altered serum Trp and Kyn (66). Trp feeding was shown to be protective in a mouse model of colitis, and another study demonstrated enhanced mucosal immunity and fungal colonization resistance in mice following expansion of indole aldehyde-producing commensal *Lactobacillus* (7, 67). Despite these promising experimental results, successful clinical trials or interventions that manipulate Trp or its microbial derivatives to benefit human disease have not developed. This could be due to a lack of understanding of the Trp-metabolizing microbes in the human microbiome, which are likely distinct from those in rodents (68). It is especially difficult to establish candidate probiotic strains as thriving members of complex the gut microbiome ecosystem, so the current paradigm of probiotic therapy could be transient at best. Currently, promising studies are exploring small molecules that act directly on host proteins such as IDO and AHR (69). A key hallmark of IBD is dysbiosis of the gut microbiome, or alteration of the abundance and distribution of gut bacteria (70, 71). The gut microbiome normally provides molecular signals such as Trp metabolites that promote mucosal homeostasis (2, 72, 73). IBD patients are shown to have altered concentrations of these Trp metabolites, highlighting the importance of these molecules in IEC barrier restitution, regulating inflammation, and maintaining GI health.

## Aryl Hydrocarbon Receptor (AHR) Recognition of TRP Metabolites

AHR is a ligand-activated transcription factor that displays promiscuous ligand-binding properties and an array of pleiotropic effects across various tissues (55, 72, 74–88). Several host and bacterial Trp metabolites are ligands for AHR, and there are polymorphisms in the AHR gene among human and other mammalian populations that result in differences in binding affinities and subsequent cellular responses to certain ligands (28, 89–92). AHR generally binds molecules that feature polyaromatic hydrocarbon (PAH) rings, making Trp derivatives a subset of many possible ligands and AHR a node in a versatile intra-kingdom communication system. For example, Trp can be converted by UV radiation in skin or chemical reactions in the GI tract to create the high-affinity endogenous AHR ligand 6-formylindolo [3,2-b]carbazole] (FICZ) (93). As mentioned earlier, gut microbe-derived Trp metabolites largely stem from the initial conversion to the AHR ligand indole, which can also be converted by microbial enzymes to a variety of other AHR ligands, either within the same bacterial cell or by exchanging among other gut microbial communities for further processing.

The structure of AHR underscores its diverse range of effects. This includes being a member of the bHLH domain family of transcription factors involved in the DNA binding and dimerization with its binding partner aryl hydrocarbon receptor nuclear translocator (ARNT/HIF1β). AHR contains two PAS domains (Period circadian protein, ARNT, and Single-minded protein, PER-ARNT-SIM) that sense environmental changes and regulate circadian rhythms through protein-protein interactions. Ligand binding causes conformational changes that expose nuclear localization signals and facilitate translocation to the nucleus. In the nucleus, AHR forms a heterodimer with ARNT and binds to the AHR response elements (a.k.a. AHRE, DRE or XRE), which consists of the general DNA consensus sequences 5’-TNGCGTG-3’ within or nearby promoters of gene targets (94). Xenobiotic-metabolizing enzymes of the cytochrome P450 family are main targets of AHR induction, including Cyp1a1 and Cyp1b1, and are responsible for the degradation and clearance of many AHR ligands, including Trp derivatives (95).

Since many Trp derivatives—host and microbial—can bind and activate AHR, understanding AHR-mediated pathways could illuminate the mechanisms of cross-kingdom communication. AHR activation at mucosal sites is generally thought to be beneficial due to the immunomodulatory role of AHR signaling and supported by the generally protective role of AHR signaling in mouse models of disease (96–99). A more detailed look into the effects of AHR activation in epithelial and immune cells is provided in the designated sections below. While animal research is invaluable to biology, studies of AHR function are sometimes confounded by the relevancy of mouse strains that are typically used. For instance, AHR knockout (*ahr* ^-/-^) mice exhibit extensive abnormalities in vascular, hepatic, skin, bladder, and hematopoietic functions (100), increased susceptibility to experimentally induced colitis (67, 98, 101, 102), and exhibit higher prevalence of intestinal tumorigenesis (103, 104). Thus, any conclusions to be made about a disease model in these animals must consider that the animals are developmentally distinct from C67Bl/6 wild-types and possibly more susceptible to disease for physiological reasons. *Ex vivo* models derived from these animals must also consider the impact that congenital defects may impart to developmental and epigenetic programs in these cells as well. One plausible solution to this would be the use of a conditional AHR knockout using promoter-driven Cre systems. The most widely used (and perhaps the only available) transgenic line that contains exons within the AHR gene flanked by loxP sites was first published in 2004 and deposited at Jackson Labs (105, 106). Development of these mice depended upon a clone derived from the AHR allele carried by 129SvJ mice (107). 129SvJ mice carry the AHR^d^ allele, which has the weakest affinity for AHR ligands such as TCDD relative to the high-affinity AHR^b^ allele present in C57BL/6 mice (four alleles of AHR have been described in laboratory mice) (108). Indeed, mice expressing different variants of the AHR allele have been reported to exhibit different responses to the same ligand (91). Although mice expressing the human AHR allele, where similarities have been drawn to the mouse AHR^d^ allele under certain contexts, has been shown to be more responsive to some tryptophan metabolites compared to the mouse AHR^b^ receptor (90, 91), the response of the mouse AHR^d^ receptor to microbe-derived tryptophan metabolites requires further investigation. Many mouse studies of AHR biology have used ubiquitous C57Bl/6 strains, and so experimental controls and subjects reflect the response of the AHR^b^ allele. However, in AHR^fl/fl^ mice crossed to Cre transgenic lines, any cell that has not undergone Cre-mediated recombination would express the low-affinity AHR^d^ allele, making that cell entirely distinct from the orthologous cell type present in ubiquitous C57Bl/6 mice in the exact gene that is under study. Thus, it is imperative that experiments do not compare Cre-expressing AHR^fl/fl^ mice with C57Bl/6 “wild-type” lines without the inclusion of Cre-deficient AHR^fl/fl^ controls. Researchers should be considerate of this and be clear in describing whether control mice are Cre-deficient littermates or genetically distinct C57Bl/6 wild types. In fact, some groups prefer to use the DBA/2 strain, which is genetically nearly identical to C57Bl/6 except for being homozygous for AHR^d^. and DBA/2 mice are healthy and lack the developmental defects present in *ahr-/-* mice. Furthermore, the behavior of any murine AHR may not necessarily reflect that of human AHR to the same ligand (91, 109, 110). These comments are not an indictment of any study, but this feature of AHR^fl/fl^ mice is not often discussed. This may present an opportunity for clarification on the role of AHR in mice and human models.

While AHR is the best–described host receptor for Trp metabolites, pregnane X receptor (PXR) is another ligand-activated transcription factor with roles in metabolism of xenobiotic and drug compounds, as well as endogenous molecules such as bile acids and steroids (111). Indole propionic acid (IPA), a bacterial Trp metabolite, was shown to interact with indole to activate PXR and induce transcription of its target enzyme CYP3A4 (112). The same study demonstrated that administration of *Clostridium sporogenes*, which produces IPA, ameliorated intestinal inflammation in a PXR-dependent fashion. IPA production by bacteria was also demonstrated to modulate vasodilation and control endothelial cell eNOS expression in a PXR-dependent manner (113). Recently, additional bacterial Trp metabolites and their analogs have been shown to activate PXR, albeit moderately (114–116). PXR has roles in several diseases and is responsible for the metabolism and clearance of many classes of drugs. Therefore, studies determining the extent that microbial metabolites can influence the PXR pathway will better elucidate mechanisms of host-microbiome metabolic cross-talk. These studies also demonstrate that the biological signaling function of Trp metabolites is not entirely AHR-dependent.

## Trp Metabolism in the Intestine and Beyond

### Intestinal Epithelial Cells

**Figure 1:**
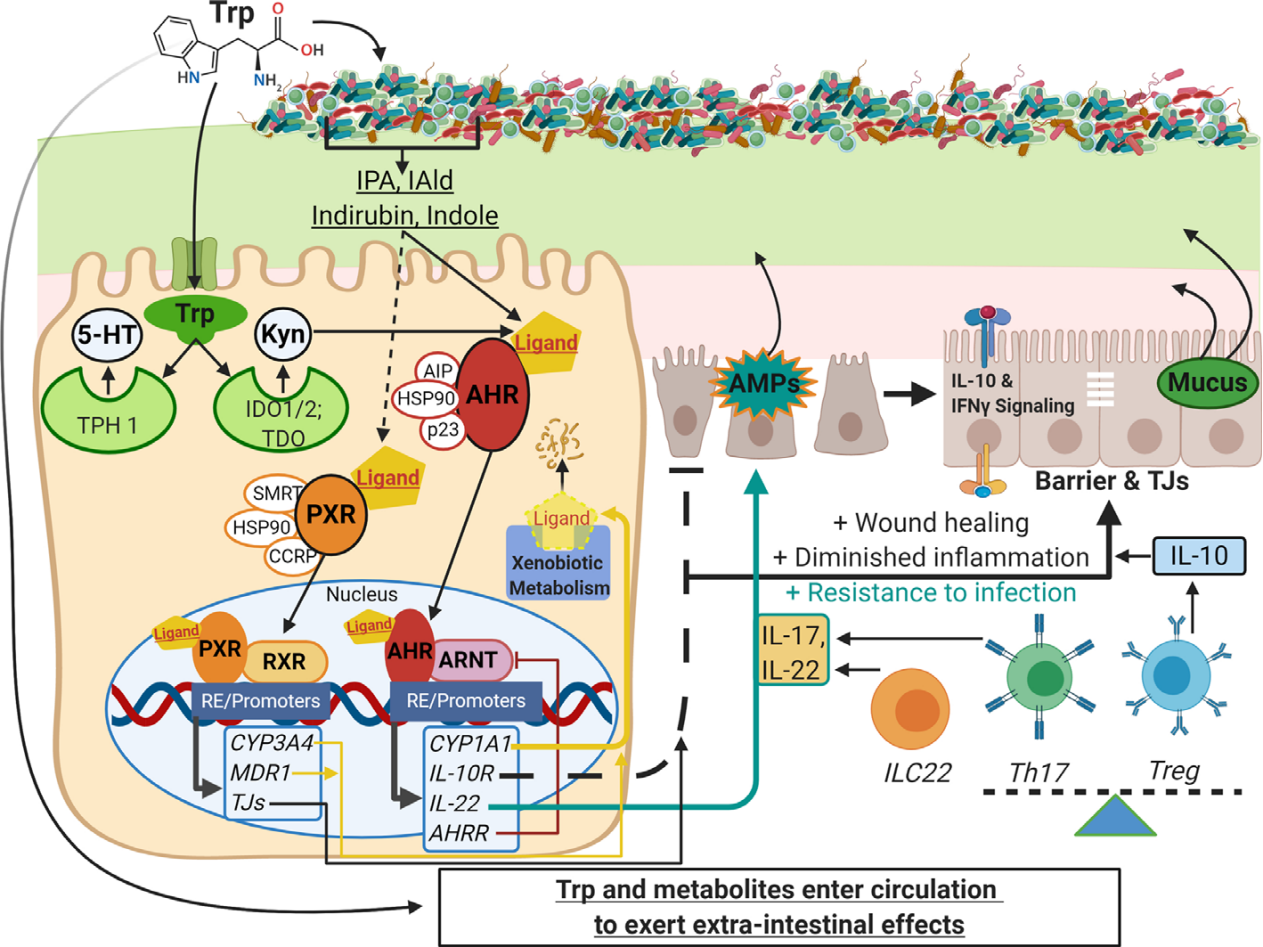
Schematic of Trp metabolism, signaling pathways, and modulation of IEC and immune cell functions. On the left side, Trp enters host cells through various amino acid transporters and is metabolized endogenously: TPH1 (or TPH2 in the periphery) is the rate-limiting enzyme in conversion to 5-HT, and IDO and TDO enzymes convert Trp into Kyn. Gut microbes synthesize Trp *de novo* and convert this essential amino acid to numerous metabolites such as IPA, IAld, and indirubin. These metabolites, as well as endogenous Trp metabolites like Kyn, are shown to bind the ligand-activated transcription factor AHR and in some cases bind the transcription factor PXR. Both AHR and PXR are bound to chaperone proteins in the cytosol and ligand binding triggers nuclear translocation, heterodimer formation with either ARNT or RXR, respectively, and regulation of gene expression through heterodimer binding of response elements on various promoters throughout the genome (RE/Promoters). Genes upregulated by AHR and PXR include xenobiotic metabolizing enzymes (yellow lines), negative regulators (e.g. AHRR, red line), and effector molecules that modulate other pathways important in barrier function, dampening inflammation, and resistance to pathogens (black and teal lines; e.g. IL-10R, IL-22, IL-17). On the right side are other pathways involving IEC and immune cell functions that are influenced by Trp metabolite signaling: TJ formation, AMP and mucus secretion, IFN-γ signaling (influences IDO1 expression, alters Kyn metabolism and IL-10R expression), and the differentiation of immune cell subsets that regulate inflammatory responses (e.g. Th17, Treg and ILC22 cells). This image was created in BioRender.com. Trp, tryptophan; IEC,intestinal epithelial cells; TPH, tryptophan hydroxylase; 5-HT, serotonin; IDO, indolamine 2, 3-dioxygenase; TDO, tryptophan 2,3-dioxygenase; Kyn, kynurenine;IPA, indole-3-propionic acid; IAld, indole-3-carboxaldehyde; AHR, aryl hydrocarbon receptor; PXR, pregnane X receptor; ARNT, AHR nuclear translocator; RXR,retinoid X receptor; AHRR, AHR repressor; TJ, tight junctions; AMP, antimicrobial peptides; Th and Treg, helper and regulatory T cells, respectively.

IECs are often considered to be front-line defenders that protect hosts from foreign luminal content and pathogens. Importantly, they serve as mediators between host and luminal content to appropriately allow the symbiosis of beneficial microbes, inhibit chronic inflammation in response to innocuous dietary antigens, and provide defensive strategies if imbalance occurs. Activation of AHR in IECs modulates a suite of phenotypic changes that promote host health (**Figure 1**). Activation of AHR promotes intestinal barrier function, resolves inflammation, ​and maintains overall mucosal homeostasis (55, 78, 117)​. The importance of AHR signaling is exemplified by numerous *in vivo* studies utilizing endogenously produced (93, 96, 103), exogenously acquired (67, 101, 118), and microbe-derived ligands (7, 9, 65, 119) to ameliorate pathologies associated with IBD and gastrointestinal infection. Multiple studies have demonstrated that Trp feeding ameliorates DSS colitis in an AHR dependent manner, and at least some of that protection can be recapitulated by feeding with microbial metabolites (67, 120). Ligands like indole and FICZ are shown to enhance IEC barrier function by regulating tight junction protein expression and distribution (121, 122). Indole acrylate produced by *Peptostreptococcus* spp can stimulate mucus production (54), and IL-22-driven antimicrobial peptide (AMP) secretion is enchanced by IAld derived from *Lactobacillus* spp (7). Dietary and endogenously produced ligands along with many microbe-derived metabolites have all been shown to decrease susceptibility to infection (7, 8, 119), reduce inflammation and ameliorate pathologies related to IBD or experimentally-induced GI injury (9, 32, 54, 74, 76, 96–99, 123–127). Additional receptors can mediate the response of IECs to Trp metabolites. SERT is expressed in high abundance by IECs to regulate the amount of available 5-HT. 5-HT receptors are expressed on epithelial cells to respond to serotonin produced by enterochromaffin cells and serotinergic neurons within the intestinal tract.

One area that remains controversial is the role of AHR signaling in colorectal and other GI cancers, since AHR is shown to have both pro- and anti- apoptotic and tumor suppressor properties (55, 128). AHR-deficient cancer models have demonstrated increased tumorigenesis while constitutive activation of AHR induces gastric tumor formation (129–131). Similarly, IDO/TDO, the rate-limiting enzyme that converts Trp to the AHR ligand Kyn, is a target for anti-cancer therapies due to its immunosuppressive functions within tumor cell environments (132). Again, elucidating how AHR signaling contributes to IEC differentiation and proliferation will further our understanding of how the scales can be tipped away from disease and towards GI health.

### Immune Cells

As mentioned previously, Trp metabolism has a potent impact on innate and adaptive immunity. IDO is upreguated in antigen presenting cells and other innate immune cell types in response to IFN-γ, which is highly expressed in the setting of inflammation. Once generated, Kyn and some of its downsteam metabolites can bind AHR on diverse cell types to alter the inflammatory response. Microbial and dietary Trp metabolites are also able to bind AHR in immune cells within and outside of the intestinal tract. AHR signaling on immune cells is typically associated with immune regulation, thus increase of exogenous or endogenous circulating AHR ligands could be critical for controlling inflammatory diseases in any tissue. AHR ligand binding can control differentiation of immune cell subsets. For instance, AHR signaling regulates the Treg/Th17 axis, which is involved in anti- and pro-inflammatory responses, respectively (55, 84, 88, 133, 134), and the differentiation of innate immune cells like dendritic cells (135) and macrophages (99, 136), which are the first responders to injury and infection. AHR activation is also shown to be necessary for the development of ILC22 cells (a subset of group 3 ILCs) that serve as an important source of IL-22 and contributes to protection against intestinal bacterial infection (7, 102).

Other immunomodulatory roles of AHR signaling include suppressing the activity of dendritic cells so that they are less able to stimulate and polarize T cells (137). IDO1 is required for lipopolysaccharide (LPS) tolerance, which is a bacterial product shown to trigger in inflammation, *via* its production of AHR ligands (119). Several inflammatory diseases show altered Trp metabolism, including changes in circulating Trp, decreased IDO, and decreased AHR expression, although whether those observations are cause or effect in disease is unclear (9, 19, 138, 139). While many recent studies have focused on IL-22 as an effector molecule in Trp metabolism and AHR activity, AHR ligands can elicit an array of antinflammatory molecules, including the upregulation of IL-10 and its receptor IL-10R1 (9, 140).

### The Central and Peripheral Nervous Systems

**Figure 2:**
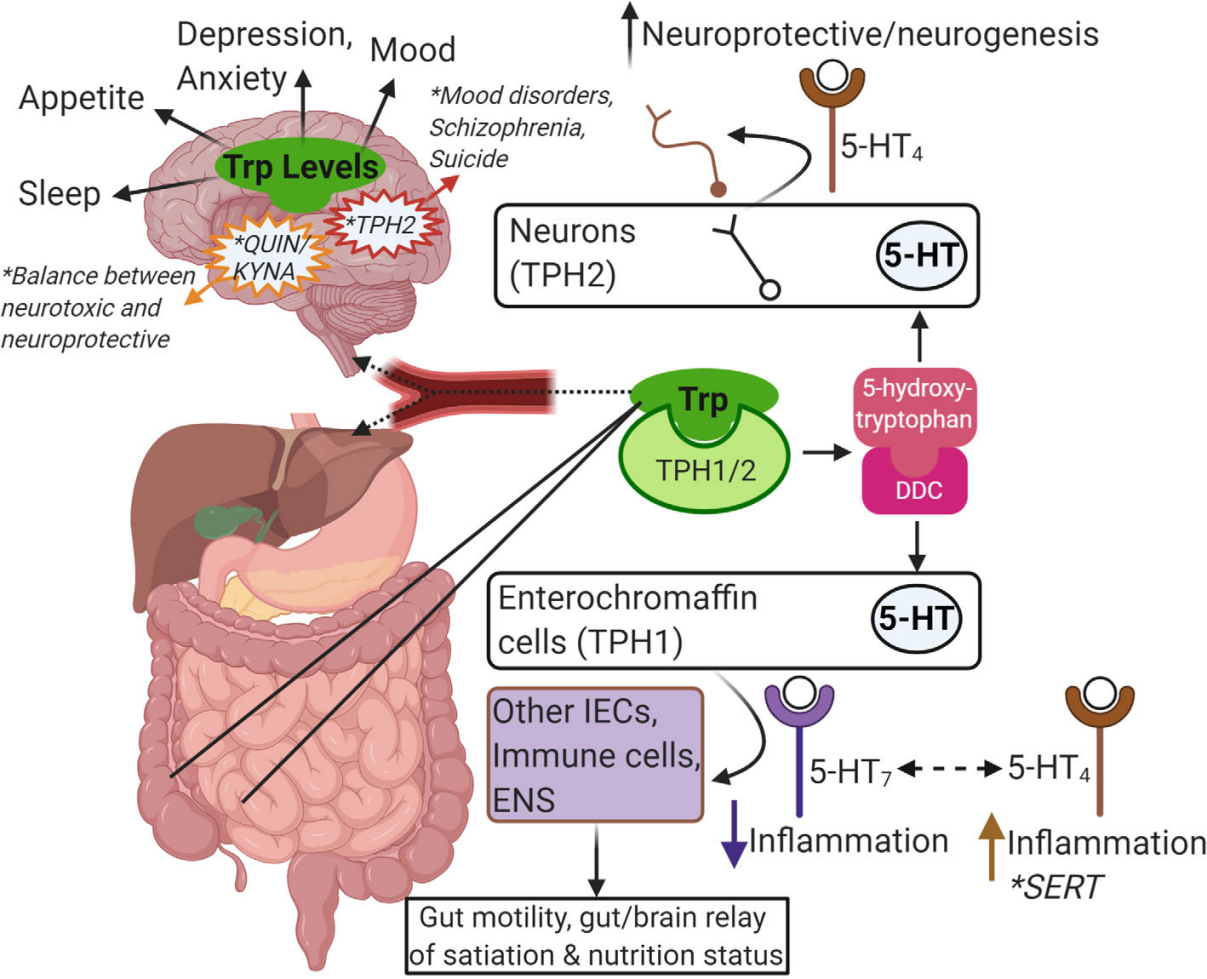
Summary of Trp-centric gut-brain axis. Trp from food or microbes is absorbed in the intestine. From there, it can be metabolized by IECs or immune cells in the intestinal mucosa, or it can enter the blood stream (as free Trp or bound to albumin). Once in the blood stream it is absorbed by other tissues in the periphery like the liver or continues to the brain through the blood-brain-barrier to get metabolized by neurons, astrocytes and glial cells. Trp is metabolized by TPH1/2 and further metabolized by DDC to serotonin (5-HT). Intestinal epithelial enterochromaffin cells are the major source of 5-HT in the gut and is important in gut motility and in communicating nutritional status to the brain *via* the ENS. Depending on the receptor, 5-HT can also impact inflammatory responses in other IECs or local immune cells. SERT is responsible for breaking down 5-HT, and reduced SERT levels has been linked to IBD and IBS. In the brain, 5-HT is important in neuroprotection and neurogenesis. In general, the levels of Trp and its metabolites are linked to many brain functions, mood, and mental health disorders (e.g. mutations in TPH2, the balance of Kyn metabolism into QUIN and KYNA). This image was created in BioRender.com. Trp, tryptophan; IEC, intestinalepithelial cells; TPH, tryptophan hydroxylase; DDC, dopa decarboxylase; 5-HT, serotonin; ENS, enteric nervous system; SERT, SLC6A4, 5-HT transporter; IBD,inflammatory bowel disease; IBS, irritable bowel syndrome; Kyn, kynurenine; QUIN, quinolinic acid; KYNA, kynurenic acid.

Dietary Trp enters the central nervous system *via* endothelial transporter proteins specific for large neutral amino acids. The amount of free Trp available to cross the blood-brain barrier *via* these transporters depends on the amount of Trp that is unbound to albumin, which is at baseline approximately 10% of the Trp in plasma (141). As seen in other tissues like the intestinal tract, once Trp crosses into the CNS, it can be applied to protein assembly, 5-HT, melatonin, and tryptamine production, or applied to the Kyn pathway (**Figure 2**). Due to the roles of 5-HT in mood, depression, anxiety, sleep, and appetite, studies have attempted to acutely alter Trp levels and determine the impact on 5-HT production and function. Trp supplementation, usually *via* increasing carbohydrates and decreasing protein, in humans and animal studies has been shown to improve mood, cognition, memory, and sleep, while acute Trp depletion inhibits those functions (142, 143). In human studies, most significant effects were seen in susceptible individuals (e.g. subjects with a history of aggression or depression) compared to individuals with no known history of such behaviors (144, 145). Mutations in *tph2*, the Trp hydroxylase gene expressed specifically in the CNS and not in the periphery, have been linked to mood disorder, schizophrenia, and increased likelihood of suicide (146, 147). Peripheral serotonin is elevated in patients with autism spectrum disorder, but in a mouse model of autism serotonin levels were decreased in the intestinal tract to promote intestinal symptoms like constipation and reduced gut transit like that seen in autistic patients (148, 149).

Kyn can cross the blood brain barrier or become synthesized in the CNS. As in all tissues, Kyn can then act as an AHR agonist or be further metabolized into quinolinic acid or kynurenic acid by two distinct pathways. Kyn is metabolized into 3-hydroxykynurenine by the enzyme KMO prior to being further metabolized into quinolinic acid, or Kyn is metabolized into kynurenic acid by KAT enzymes. Quinolinic acid is neurotoxic due to its role as a NMDA agonist and because it promotes glutamate release and inhibits glutamate reuptake (150). Quinolinic acid is found at high concentrations in the CNS of patients with ALS, which is also linked to glutamate toxicity (151). On the other hand, kynurenic acid is neuroprotective due to its role as a NMDA antagonist and may inhibit glutamate toxicity (152). NMDA antagonism by kynurenic acid is thought to be a mechanism for recovery and protection from stroke, epilepsy, and Huntington’s disease (153–155). Interestingly, kynurenic acid has been also shown to be an AHR ligand and likely exerts anti-inflammatory effects in the CNS and periphery *via* pathways discussed above. IDO1 is highly expressed in patients with autism spectrum disorder, and autistic patients have a build-up of quinolinic acid but low levels of kynurenic acid in the CNS (156). While Kyn levels are typically thought to be protective, an increase in Kyn was observed in rats that received fecal microbiome transplants from depressed donors as well as the donors themselves. In another study, mice subject to chronic stress had an increase in IDO1 expression that could be ameliorated by a probiotic that inhibited IDO1 function (157, 158). These studies offer evidence that Trp metabolism may be imbalanced away from serotonin in mood and depressive disorders.

Trp metabolism in the CNS is the result of multiple cell types. While neurons are the primary serotonin producers in the CNS, glial cells have a major role in generating and responding to bioactive metabolites from the Kyn pathway (159). Microglia and astrocytes, as well as infiltrating and immune cells, can express IDO1. Microglia that lack AHR promote autoimmune demyelination *via* their role in promoting astrocyte-driven inflammation. This same study also showed that Trp depletion exacerbated disease, which could be rescued by the administration of indoxyl-3-sulfate, a derivative of bacterially generated indoles (160, 161).

Several studies have demonstrated the impact of Trp metabolites on the enteric nervous system (ENS) and the cross-talk between intestinal epithelial and immune cells, the ENS, and the CNS—the gut-brain axis. Due to the constant influx of foreign elements into the lumen, the intestinal tract must constantly sense stimuli to generate sympathetic and parasympathetic responses. The intestinal tract is innervated extrinsically by vagal and pelvic neurons and intrinsically by local neurons located in the submucosal plexus and the myenteric plexus of the small and large intestines. Intestinal epithelial and immune cells respond to nutrients, microbial metabolites, and physical factors to produce neuroactive molecules including hormones, neuropeptides, and small metabolites. These molecules can signal onto extrinsic and intrinsic neurons to promote or regulate local and peripheral reflexes such as peristalsis, secretion, and hunger. Trp metabolites produced by host and microbial cells can influence the ENS directly or indirectly. 5-HT production in the gut and neuronal responses to 5-HT is a prominent example of the impact of Trp metabolism in the intestine. Epithelial cells, specifically enterochromaffin cells (ECs), express TPH1 to generate 5-HT that acts locally and distally on multiple cell types including the ENS (162). 5-HT can also be generated by the neurons in the ENS *via* TPH2, which is critical for normal ENS development and functions like motility (163). 5-HT is released following food intake and gastrointestinal distention to stimulate peristalsis and gut motility and relay messages between the ENS and the CNS (164–166). Serotonergic neurons also promote epithelial cell homeostasis by engaging the receptor 5-HT_2A_ on enteric cholinergic neurons in the myenteric plexus, which may then produce signals to promote epithelial cell proliferation (167). 5-HT signaling has complicated roles in intestinal inflammation: One study found that 5-HT signaling was reduced in IBD (notably in patients suffering from both IBD subtypes, ulcerative colitis and Crohn’s disease), though it’s difficult to parse whether this is due to inflammation-mediated loss of enterochromaffin cells or whether the loss of 5-HT production can influence or be influenced by inflammatory processes (168). The role of 5-HT and its signaling onto enteric neurons has a strong link to IBS. IBS is similar to IBD in that the etiology involves a combination of genetic, environmental, and gut microbial factors. IBS is characterized by aberrant gut motility and visceral hypersensitivity that collectively contribute to bouts of diarrhea and constipation and heightened pain perception during digestion and bowel movements (169) IBS patients may express lower levels of SERT, and certain SERT alleles may present an increased risk for developing IBS and other 5-HT-related pathologies, though this finding is inconsistent across studies (29, 170, 171). There is also reported link between IBS and increased levels of 5-HT, possibly as a result of dysfunctional SERT (172, 173). 5-HT acts on neurons *via* the receptors 5-HT_3_ and 5-HT_4_. 5-HT_3_ signaling activates intrinsic and extrinsic neurons to promote secretion and motility, thus 5-HT_3_ antagonism is an attractive therapy in diseases like IBS (174). Similarly, 5-HT_4_ binding increases gastric motility and acetylcholine release, which promotes epithelial cell secretory pathways; 5-HT_4_ agonists could provide therapy to IBS patients with chronic constipation (175, 176). Interestingly, 5-HT_4_ also promotes neuron survival and development and inhibits pain perception in the intestine (28, 177, 178). Altogether, inhibitors of 5-HT receptors and SERT, including selective serotonin reuptake inhibitors (SSRIs) have been studied and reported on extensively for therapeutic interventions for treating the various presentations of IBS (179–181). Additionally, promising data has been published to demonstrate a TPH inhibitor that is unable to cross the blood brain barrier could improve IBS symptoms without influencing 5-HT signaling in the CNS (182). Perhaps other mechanisms to regulate 5-HT signaling *via* dietary or microbial interventions of Trp metabolism could result in additional possibilities.

The ENS, like other neurons, can be influenced by additional Trp metabolites. The microbiome can stimulate expression of AHR in enteric neurons, and AHR activity promotes enteric neuron function and intestinal peristalsis (183). Limited studies exist that explore the effects of quinolinic acid and kynurenic acid on enteric neurons (184). Excitatory NMDA receptors are responsible for peristalsis and nociception, and intestinal inflammation has been linked to their upregulation in animal models (184–186). Kyneurinic acid or manipulation of the balance between kynurenic acid and quinolinic acid could inhibit these pathways to limit motility and pain and reduce glutamate toxicity in enteric neurons during inflammation.

## Concluding Remarks and Future Considerations

Elucidating the metabolic currency exchanged between the diet, the intestinal microbiota and the host is crucial for the development of new therapeutics to modulate intestinal microbial dysbiosis and maintenance of tissue homeostasis, both locally and systemically. Our comprehension of the interactions between host- and microbiota-mediated Trp metabolism, mucosal tissue homeostasis, and host immunity has grown immensely over the past decade. For instance, the role of Kyn, the predominant endogenous Trp metabolite, is well established as a potent mediator of host inflammatory responses, exerting effects on a number of immune cells through AHR signaling. While much of this work has been gut-centric, it has become clear that the endogenous Trp metabolites impact many extra-intestinal tissues including the nervous system. Thus, host metabolism of dietary or microbially produced Trp has both systemic and pleiotropic implications. Expansion of our understanding of these pathways will have important implications for the potential treatments of a number of human ailments.

While the scientific community has made great strides in elucidating the influences of endogenous Trp metabolism on host health, our understanding of microbial Trp catabolism, both within the microbiome and its impact on the host, is less advanced. Studies have demonstrated that Trp availability and metabolism impact both structure and function of the microbiome. Additionally, it is clear that a number of bacteria can generate Trp metabolites including indoles and an array of indole-containing compounds. These microbial Trp derivatives, both directly and indirectly, have critical roles in the regulation of tissue homeostasis and host immune responses through activation of host AHR and PXR proteins. Examples include modulation of intestinal epithelial barrier and mediation of host immune tolerance of the gut microflora. Furthermore, many microbial-derived Trp metabolites have been identified in circulation, indicating the ability to exert systemic effects. However, there remains much work to do to expand our understanding of the microbiota members that contribute to Trp metabolism, the breadth of Trp metabolites produced, and the impact of these molecules on the host.

It is enticing to envision potential therapeutics arising from these studies involving perturbation of Trp metabolic pathways or Trp metabolite administration for a number of human disease states, including inflammatory and metabolic diseases, depression and mood disorders, as well as cancer. However, there is much more work to be done to increase our understanding of the mechanistic underpinnings of Trp metabolism and the modulation of these dynamic pathways. These future studies must take care to recognize the limitations of existing *ex vivo* and *in vivo* model systems as new models are developed that will enable a more complete elucidation of these metabolic pathways.

## Author Contributions

All the authors contributed extensively to the work presented in this manuscript. HG-W and BRJ performed the literature search and wrote the manuscript. DK revised the manuscript. All authors contributed to the article and approved the submitted version.

## Funding

We want to acknowledge all of our funding sources and grant support: National Institute of Diabetes and Digestive and Kidney Diseases of the National Institutes of Health under award number R01DK09945; National Institute of General Medical Sciences of the National Institutes of Health under award number U54GM115371; the United States Department of Agriculture National Institute of Food and Agriculture Hatch Project 1015883; graduate stipend support from the Molecular Biosciences Program at Montana State University (MSU); graduate research funding from the Cole-Tierney Award (MSU).

## Conflict of Interest

The authors declare that the research was conducted in the absence of any commercial or financial relationships that could be construed as a potential conflict of interest.
